# A Novel, Small Cysteine-Rich Effector, RsSCR10 in *Rhizoctonia solani* Is Sufficient to Trigger Plant Cell Death

**DOI:** 10.3389/fmicb.2021.684923

**Published:** 2021-08-23

**Authors:** Xianyu Niu, Guijing Yang, Hui Lin, Yao Liu, Ping Li, Aiping Zheng

**Affiliations:** ^1^College of Agronomy, Sichuan Agricultural University, Chengdu, China; ^2^Rice Research Institute, Sichuan Agricultural University, Chengdu, China; ^3^Key Laboratory of Sichuan Crop Major Disease, Sichuan Agricultural University, Chengdu, China

**Keywords:** *R. solani* AG1IA, SCR secreted protein, cell death, *N. benthamiana*, immune response, protein interaction

## Abstract

The necrotrophic phytopathogen *Rhizoctonia solani* (*R. solani*) is a fungus that causes disease in a wide range of plant species. Fungal genomes encode abundant, small cysteine-rich (SCR) secreted proteins, and the probable importance of these to pathogenesis has been highlighted in various pathogens. However, there are currently no reports of an *R. solani* SCR-secreted protein with evidential elicitor activity. In this study, the molecular function of 10 SCR-secreted protein genes from *R. solani* was explored by agroinfiltration into *Nicotiana benthamiana* (*N. benthamiana*) leaves, and a novel SCR protein RsSCR10 was identified that triggered cell death and oxidative burst in tobacco. RsSCR10 comprises 84 amino acids, including a signal peptide (SP) of 19 amino acids that is necessary for RsSCR10 to induce tobacco cell death. Elicitation of cell death by RsSCR10 was dependent on Hsp90 but not on RAR1, proving its effector activity. Two cysteine residues have important effects on the function of RsSCR10 in inducing cell death. Furthermore, RsSCR10 showed cross-interaction with five rice molecules, and the inferred functions of these rice proteins suggest they are instrumental in how the host copes with adversity. Overall, this study demonstrates that RsSCR10 is a potential effector that has a critical role in *R. solani* AG1 IA-host interactions.

## Introduction

Necrotrophic diseases negatively affect the production of numerous species of crops, vegetables, fruit trees, and pastures, including the major food staples in the world—rice, corn, and wheat (Sneh et al., 1991; Staats et al., 2005; Michielse and Rep, 2009). A basidiomycete species complex, *Rhizoctonia solani* is a phytopathogen that exhibits a broad host range and is problematic for farmers, particularly in tropical areas with high humidity (Ogoshi, 1987; Taheri and Tarighi, 2011). *R. solani* encompasses necrotic cell death on aerial plant tissues, and its lifestyle is a threat to host plants due to its invasion (Singh et al., 2019). *R. solani* releases various molecular proteins, such as phytotoxins, carbohydrate-active enzymes, and effector proteins, during infection of plant hosts to achieve colonization. Among these proteins, effectors in *R. solani* are the least studied. Numerous candidate effector molecules have been predicted in the genome and transcriptome sequences of *R. solani* (Zheng et al., 2013; Hane et al., 2014; Wibberg et al., 2014, 2016; Anderson et al., 2017; Ghosh et al., 2018), but the number of functional effectors is considerably less, and their molecular functions are largely unknown.

SCR-secreted proteins have attracted much attention in plant pathology (Stergiopoulos and De Wit, 2009) because this type of protein is the source of elicitors. Typically, SCR-secreted proteins of phytopathogens are identified by their short length of 50–300 amino acid residues, high (3% or more) cysteine content, presence of a signal peptide (SP) sequence, and absence of transmembrane domain (Saunders et al., 2012; Lu and Edwards, 2015; Anderson et al., 2017). Cysteine residues of SCR-secreted proteins form internal disulfide bonds (Overgaard et al., 2003), which are believed to enhance the stability of the proteins against proteases in the plant apoplast (Kamoun, 2006). Approximately 100 or more SCR-secreted proteins have been predicted in each genome of various phytopathogens including species of the genus *Fusarium*, *Ustilago maydis*, and *Magnaporthe oryzae* (*M. oryzae*) (Mueller et al., 2008; Dong et al., 2015). Several SCR-secreted proteins were characterized as phytopathogenic factors, effectors, pathogen-associated molecular patterns (PAMPs), or avirulent elicitors (Liu et al., 2016; Dagvadorj et al., 2017; Nie et al., 2019). A few hundred SCR proteins were predicted in *R. solani* genomes: 103 genes in anastomosis group 1 (AG-1) subgroup IA (Zheng et al., 2013) and 308 genes in AG-8 (Hane et al., 2014). Our recent study involved a pre-test of a few SCR-secreted proteins from *R. solani* AG1 IA (Xia et al., 2017), but there is currently no evidence validating the importance of SCR-secreted proteins in the pathogenicity of *R. solani*.

Invasion patterns, including microbe-associated molecular patterns (MAMPs), PAMPs, effectors, and (*a*)virulent elicitors, are involved in the primary process of phytopathogen-plant host interaction (Jones and Dangl, 2006). To enhance understanding of the pathogenic strategy and underlying molecular mechanisms in the phytopathogen *R. solani*, identification of such pathogenic molecules is the vital initial step. Furthermore, the discovery of protein cross-interactions between invasion patterns and plant host proteins may indicate how *R. solani* interferes with host cell defense reactions and physiological activities. Consequently, the current study aimed to identify SCR-secreted proteins that were possible effectors of *R. solani*, using the technique of agroinfiltration-based transient bioassay in *Nicotiana benthamiana*. A novel gene, *RsSCR10*, encoding a potential SCR-secreted protein was identified and characterized. Gene regulation, subcellular trafficking behaviors, and protein cross-interaction experiments demonstrated that RsSCR10 is an important pathogenic molecule of *R. solani*.

## Materials and Methods

### Fungal Materials

The national standard strain of *R. solani* AG1 IA was precultured in potato sucrose agar (PSA) medium (per liter: 200 g boiled potato extract, 20 g sucrose, and 15 g agar powder), and then small pieces of agar block with fresh *R. solani* mycelia were transferred into potato sucrose broth (PSB) medium (200 g/L boiled potato extract and 20 g/L sucrose) to amplify the mycelia. Mycelia were collected after 2 days and immediately frozen using liquid nitrogen then stored at −80°C until use.

### Plant Materials

Seeds of *N. benthamiana* and *Arabidopsis* were sown on soil and placed in a chamber at 25°C with a 16-h illumination cycle per day under 60–80% humidity. After 2 weeks, tobacco seedlings were used for virus-induced gene silencing (VIGS) experiments. Mature leaves of 4-week-old plants were used for effector bioassays and gene expression analysis. Sterilized seeds of the rice cultivar Nipponbare were sown on half-strength Murashige Skoog medium (Murashige and Skoog, 1962) supplemented with 2% sucrose, 1% agar, and vitamins and placed in a chamber under dark conditions at 25°C. After approximately 2 weeks, rice leaves were used for protoplast preparation. *Oryza sativa* subsp. *indica* R600, a rice variety applied by Sichuan Agricultural University and certified by China, is classified as a moderately susceptible variety following inoculation with *Magnaporthe grisea* (the rice blast fungus). R600 was cultivated in the summer season in a rice paddy field of the Wenjiang experimental field of Sichuan Agricultural University, located in Huihe village (Chengdu, Sichuan Province, China; 104.06°E, 30.67°N). Before rice plants reached the tilling stage, leaf blades were detached, and *R. solani* AG1 IA mycelia were inoculated on the surface of the detached leaves for gene expression assays.

### Total RNA Preparation

*R. solani* mycelia were powdered in liquid nitrogen using a mortar and pestle, then total RNA was isolated using a Spin Column Fungal Total RNA Purification Kit (Sangon, Shanghai, China) according to the manufacturer’s protocol. Tobacco and rice tissues were also powdered in liquid nitrogen using a mortar and pestle, and then total RNA was extracted using a Spin Column Plant Total RNA Purification Kit (Sangon) according to the manufacturer’s instructions.

### cDNA Cloning

cDNAs of candidate SCR effectors were amplified from *R. solani* AG1 IA mycelia by RT-PCR. Briefly, the first-strand cDNA was synthesized using a Transcriptor First Strand cDNA Synthesis Kit (Roche, Basel, Switzerland) according to the manufacturer’s protocol, and cDNA fragments were then amplified using Phusion High-Fidelity DNA Polymerase (New England Biolabs, Ipswich, MA, United States) and gene-specific primers (Supplementary Table 1). The resulting PCR products were cloned into a T-vector, and the cDNA inserts were sequenced.

### Bioassay by Agroinfiltration

For validation of effector candidates, full-length coding regions of the cDNAs were directionally subcloned into the *Bam*HI and *Sal*I site of the GATEWAY binary vector pMDC32 (Curtis and Grossniklaus, 2003) using ClonExpress II One Step Cloning Kit (Vazyme Biotech Co., Ltd, Nanjing, China). pMDC32 allows overexpression of a cloned gene in plant cells driven by a CaMV35S promoter. In these constructs, 2 × FLAG peptide tags (DYKDDDDK) were fused downstream of the inserted gene by a linker peptide, allowing immune detection of transiently expressed proteins. For the SCR gene *RsSCR10*, a deletion constructs *RsSCR10*^Δ*SP*^ (lacking the SP region) was generated using another primer set (Supplementary Table 1) and was used for functional analysis of the SP. An overexpression construct of the green fluorescent protein (GFP) acted as a negative control, and both a mouse proapoptotic Bcl-2 family member (*BAX*) and *INF1* constructs were prepared as positive controls.

Constructed plasmids were transformed into *Agrobacterium tumefaciens* GV3101 using the freeze-thaw method (An et al., 1989). *Agrobacterium* clones harboring the plasmid constructs were cultured in YEP medium (0.5% yeast extract, 1% tryptone, and 1% NaCl) overnight at 28°C, and bacterial cultures were harvested and resuspended in infiltration buffer [10 mM MES-Tris (pH 5.6), 10 mM MgCl_2_, and 100 μM acetosyringone] at an optical density of 600 nm (OD_600_) of 0.5. The *Agrobacterium* solutions were pressure infiltrated into tobacco leaves using needleless syringes. A total of 20 leaves from different plants were infiltrated for each control. Cell-death status on tobacco and *Arabidopsis* leaves were monitored and photographed at 3–5 days after agroinfiltration (Wei et al., 2020).

### Site-Directed Mutagenesis

According to prediction results from the website https://predictprotein.org/, RsSCR10 has 10 cysteine residues that can form five disulfide bonds. We used splicing overlap extension PCR for site-directed mutagenesis. Two DNA fragments of *RsSCR10* were amplified from the cDNA of *R. solani* AG1 IA, and the PCR product was cloned into 35S-PMDC32 vectors. The primer sequences used were listed in Supplementary Table 1.

### Trypan Blue Staining, Oxygen Burst, and Callose Deposition Detection

For detection of dead cells, tobacco leaves were stained with trypan blue (Fernández-Bautista et al., 2016). Briefly, agroinfiltrated leaves at 5–7 days postinfiltration (dpi) were collected and immersed in ethanol: glacial acetic acid solution (3:1, *v*/*v*) for 24 h to fix the cell tissues and destain chlorophyll. The treated leaves were then soaked in a solution containing trypan blue dye [10 ml lactic acid, 10 ml glycerin, 10 ml deionized water, 10 g phenol, and 20 mg trypan blue (Sigma-Aldrich, St. Louis, United States)] for 24 h. Tissue images were taken and decolorization was performed. Samples were decolorized in 1.25 g/ml chloral hydrate solution for 3 days. Triplicated bioassays were performed to confirm the reproducibility of the effector function. Oxygen burst status in tobacco leaves was monitored by detecting accumulation of H_2_O_2_ using 3,3′-diaminobenzidine (DAB) according to the method of Thordal-Christensen et al. (1997). Briefly, agroinfiltrated tobacco leaves were detached at 72 h postinfiltration (hpi), soaked in 1 mg/ml DAB solution, and incubated at 25°C for 8 h. Leaf tissues were then boiled in 95% ethanol until all chlorophyll pigments were entirely bleached. For further removal of the background color, the bleached samples were soaked in 1.25 g/ml trichloroacetic aldehyde, and the leaves were photographed. Callose deposition was monitored at 48 h after agroinfiltration of *N. benthamiana*. All experiments were repeated at least three times.

### Immunoblotting

Frozen tobacco leaves were ground in liquid nitrogen using a mortar and pestle, and 1 g powdered sample was mixed with 2 ml extraction buffer [50 mM Tris-HCl (pH 7.4), 150 mM NaCl, and 1% Tergitol-type NP-40] containing fresh “plant-specific protease inhibitor mixture” at 1 mM final concentration and 1 mM phenylmethylsulfonyl fluoride. The sample mixture was placed in an ice bath for 30 min with vortexing every 10 min. After ultrasonication for 5 min, the sample was centrifuged at 12,000 × *g*, 4°C for 20 min, and the supernatant was collected as a crude extract. Soluble protein concentration was determined with the Easy II Protein Quantitative Kit (BCA) TransGen Biotech Co., Ltd, Beijing, China) and 150 μg soluble protein was then denatured by urea and electrophoresed in a urea polyacrylamide gel. Proteins in the gel were electrotransferred onto a polyvinylidene fluoride membrane, and the FLAG tag of transiently expressed proteins was detected using an anti-FLAG antibody (ABclonal, Inc., Wuhan, China). The immunoblotting signal was detected using chemiluminescence technology (Vigorous Biotechnology Beijing Co., Ltd., Beijing, China) and X-ray film exposure to obtain an autoradiograph.

### Virus-Induced Gene Silencing Assays in Tobacco

The tobacco rattle virus (TRV)-mediated method of gene silencing (Liu et al., 2002) uses two types of expression vector: the pTRV1 binary vector, which harbors an expression cassette of a cDNA clone of TRV RNA1, and the pTRV2-LIC-based vector, in which the target genes to be silenced are inserted via the multicloning site. These binary vectors were purchased from BioVector NTCC Inc. (Beijing, China), and cDNA clones of *RAR1* and *Hsp90* in *N. benthamiana* (GenBank ID: LC314308 and KT726860, respectively), *NbPDS* (GenBank ID: EU165355), and *GFP* were cloned into the pTRV2 vector. pTRV1 and the pTRV2-LIC-based vectors were transformed into *A. tumefaciens* GV3101. Overnight cultures of transformants for these constructs were prepared, and pTRV1 and one of the pTRV2-LIC-based clones of *Agrobacterium* cells were collected and mixed with MMA buffer [10 mM MgCl_2_, 10 mM MES-Tris (pH 5.6), and 100 μM acetosyringone] to an OD_600_ of 0.5. After incubation at room temperature for 3 h, the resultant bacterial mixture was injected into a few primary leaves of tobacco using a needle-free syringe. Three weeks later, *Agrobacterium* transformed with pMDC32-based constructs for *RsSCR10* from *R. solani* AG1 IA or *INF1* from *Phytophthora cactorum* (Genbank ID: KF893299) were infiltrated onto at least three leaves of tobacco plants. A total of 27 leaves from different plants were infiltrated for each control. Gene silencing status was verified by real-time quantitative RT-PCR (qRT-PCR).

### Subcellular Localization in Tobacco Cells

Full-length *RsSCR10* cDNA was subcloned into 2^×^35S: pHB-YFP digested with *Hin*dIII and *Bam*HI to generate the construct pHB-YFP-*RsSCR10*. This plasmid was separately transformed into *A. tumefaciens* GV3101. Agroinfiltrated tobacco leaves were excised into small pieces and imaged using a confocal laser scanning microscope (Nikon A1; Tokyo, Japan). Excitation and emission wavelengths for yellow fluorescent protein (YFP) were set at 514–527 nm.

### Cotransfection Onto Rice Protoplasts

Cotransfection assays in rice protoplasts were performed as previously described (Zhang et al., 2011; Chen et al., 2014). Namely, rice protoplasts were isolated and transfected using a plant protoplast preparation and transformation kit (Real-Times Biotechnology Co., Beijing, China) according to the manufacturer’s protocol. The pHB-YFP-*RsSCR10* plasmid was purified from *Escherichia coli* clones using an E.Z.N.A. FastFilter Plasmid Maxi Kit (Omega Bio-tek, Inc., Norcross, GA, United States) and used for polyethylene glycol (PEG)-mediated transfection.

### Function Validation of the RsSCR10 Signal Peptide

A functional test of the SP of RsSCR10 was performed using the method of Jacobs et al. (1997). Namely, the cDNA fragment corresponding to the predicted SP of *RsSCR10* was amplified using a gene-specific primer set (Supplementary Table 1) and subcloned into the vector pSUC2 (BioVector NTCC Inc.), which generated an in-frame fusion with an invertase gene. The resultant construct *pSUC2-RsSCR10^*SP*^* was transformed into the yeast *Saccharomyces cerevisiae* (*S. cerevisiae*) YTK12, and positive selection was performed on CMD-W medium, which lacks tryptophan. Positive colonies were inoculated on plates of YPRAA media for invertase secretion. YTK12 transformed with *pSUC2-Avr1b^*SP*^* was used as a positive control, while YTK12 transformed separately with *pSUC2-Mg87^*SP*^* or the empty pSUC2 vector were used as negative controls.

### qRT-PCR

*R. solani* AG1 IA was inoculated onto leaf blades of R600, and mycelia were collected at 12, 24, 36, 48, and 60 hpi. Four-week-old tobacco plants were subjected to agroinfiltration as described above, and infiltrated leaves were harvested after 12, 24, 36, 48, and 72 hpi. Tobacco plants under the VIGS experiments were maintained for an additional 3 weeks, then primary leaves were harvested for quantitative gene expression analysis.

qRT-PCR was performed using a previously described method (Wu et al., 2010) and SYBR premix ex Taq (TaKaRa Co., Ltd., Tokyo, Japan). Real-time PCR reactions were conducted using the advanced Universal SYBR Green Supermix (Bio-Rad, Hercules CA, United States). cDNAs were diluted 10-fold, and 1 μl of each dilution was used for one assay. Gene-specific primers were designed for *actin*, *NbPR1b* (Genbank ID: X17680), *NbPR2b* (GenBank ID: M60460), *NbLOX* (GenBank ID: KC585517), and *ethylene-responsive factor 1* (*NbERF1;* GenBank ID: GQ859157) in *N. benthamiana* and *RsSCR10* and the 18S ribosomal RNA (rRNA) gene in *R. solani* (Supplementary Table 1). All PCR products were confirmed to be a single peak in melting curve analyses. *Actin* and the 18S rRNA gene were used as internal references to determine relative expression levels of genes by the ΔΔCT method (Livak and Schmittgen, 2002). qRT-PCR assays included biological triplicates and technical quadruplicates.

### Yeast Two-Hybrid Assays

A normalized rice leaf cDNA library was constructed for yeast two-hybrid assays. Total RNA was extracted using TRIzol (Invitrogen Corp., Carlsbad, CA, United States), and poly (A)^+^ RNAs were purified using an Oligotex mRNA kit (Qiagen GmbH, Geschäftsführer, Germany). The integrity of the RNA fractions was analyzed by agarose gel electrophoresis. cDNAs were normalized, then ligated into the plasmid pGADT7 and transformed into the yeast *S. cerevisiae* Y2H gold strain. Total plasmids of the cDNA library were extracted for the screening of interacting proteins of interest. *RsSCR10* cDNA was subcloned into the bait vector pGBKT7, cotransformed with the rice cDNA plasmids into Y_2_H gold, and cultured on a synthetic dropout medium without leucine and tryptophan (SD/-Leu/-Trp) at 28°C for 3–5 days. Amplified yeast clones were transferred onto the synthetic dropout medium supplemented with aureobasidin A and X-α-Gal but lacking leucine, tryptophan, adenine, and histidine (SD/-Leu/-Trp/-Ade/-His/ + Aba/ + X-α-Gal) at 28°C for 3–5 days. Every colony obtained was then analyzed by PCR to determine the insert sequence. After repeating the screening at least six times, 30 proteins demonstrated a reproducible interaction with RsSCR10 and were collected as positive clones. These positive clones were separately tested in the yeast two-hybrid system to retain five candidate clones.

### Bimolecular Fluorescence Complementation

The cDNA insert of *RsSCR10* was subcloned into the vector pXY106 (N-terminal split-nYFP) (Tao et al., 2019), which contains the partial coding sequence of YFP, and this subcloning yielded the fusion protein gene YN-*RsSCR10*. Full-length cDNAs of five rice genes without the stop codon were separately cloned into the pXY104 (C-terminal split-cYFP) vector from Tao et al. (2019), generating YC-*OsP21*, YC-*PsbO*, YC-*OsHyPRP8*, YC-*OsALDH2B1*, and YC-*OsBcat-3*. The different pairs of plasmids containing the split nYFP- and cYFP-tagged protein were transiently coexpressed in leaf epidermis cells of *N. benthamiana* by using the method with agroinfiltration described above. A total of 45 leaves from different plants were infiltrated for each control. YFP signals were detected using a Nikon A1 confocal scanning microscope after 48 h of agroinfiltration.

### Luciferase Complementation Assays

The cDNA fragments of *RsSCR10* and five rice genes were subcloned into the *Sac*I/*Sal*I site of pCAMBIA1300-nLuc or the *Kpn*I/*Bam*HI site of pCAMBIA1300-cLuc (Chen et al., 2008). The resulting recombinant plasmids were, respectively transformed into *A. tumefaciens* GV3101 and used for agroinfiltration into leaves of 1-month-old tobacco plants. A total of 45 leaves from different plants were infiltrated for each control. Leaf samples were harvested within 48–72 h after agroinfiltration and smeared with a solution of D-Luciferin sodium salt (BioVision Inc., San Francisco, CA, United States) to detect fluorescence by luciferase activity.

### Prediction of Effector Proteins, SPs, and Subcellular Localization

Prediction of effector proteins was performed using EffectorP 2.0 (Sperschneider et al., 2018). Prediction of SP sequences was performed using SignalP 4.1 (Petersen et al., 2011) with the default setting parameters. Subcellular localization of rice proteins was predicted using TargetP version 1.1 (Emanuelsson et al., 2000).

### Statistical Analyses

Student’s *t*-tests were performed using Microsoft Excel software. The R statistical package^1^ was used for checking the equality of variance and conducting a two-way analysis of variance (ANOVA).

## Results

### An SCR Secreted Protein in *R. solani*, RsSCR10, Caused Cell Death

**FIGURE 1:**
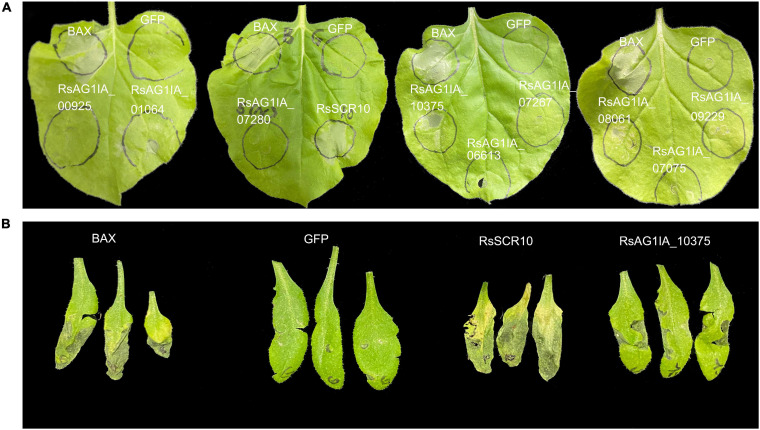
Induction of cell death in leaves by agroinfiltration of RsSCR10. **(A)** Transient expression assays of 10 putative *R. solani* effectors revealed that two candidate effectors could cause cell death or mottling symptoms in *N. benthamiana* leaves. *GFP* and *BAX* constructs were infiltrated as a negative and positive control, respectively. Representative photos were taken 4 days postinfiltration. **(B)** Cell death in *Arabidopsis* leaves. Data are the means from three independent experiments.

In a previous study on the *R. solani* genome (Zheng et al., 2013), a total of 103 SCR-secreted proteins were predicted as potential effectors. The present study used a machine-learning method for the prediction of effector proteins (Sperschneider et al., 2018) to select 10 SCRs to focus on (Supplementary Table 1). cDNAs for these genes were successfully cloned from the national standard strain of *R. solani* AG1 IA, and their molecular functions were examined by agroinfiltration-based bioassays in model plant *N. benthamiana.* Infiltration of tobacco leaves with two of these genes, *AG1 IA*_09956 and *AG1 IA*_10375, resulted in the observation of cell death with transiently expressed protein in the agroinfiltrated leaves (Figure 1A). To determine whether these two genes induced the defense response in other plants, the two constructs were transiently expressed into *Arabidopsis* leaves by agroinfiltration-based bioassays and the phenotype was examined (Figure 1B). Only AG1 IA_09956 resulted in the cell death phenotype in the non-host plant *Arabidopsis* leaves (Figure 1B). Hence, the cloned cDNA of *AG1 IA* _09956 was designated *RsSCR10* and was a transcript variant from the gene *AG1 IA_09956*. Consequently, RsSCR10 was selected as the object of subsequent research. There was more accumulated hydrogen peroxide (H_2_O_2_) and callose deposition in the agroinfiltrated leaves than in control leaves (Supplementary Figure 1), accompanied by distinct gene expression patterns for the effector compared with the GFP control (Supplementary Figure 2). These findings suggested that RsSCR10 stimulated basal host immunity in tobacco, resulting in oxidative burst and callose deposition.

### RsSCR10 Is Secreted *via* the Classical Secretory Pathway

**FIGURE 2:**
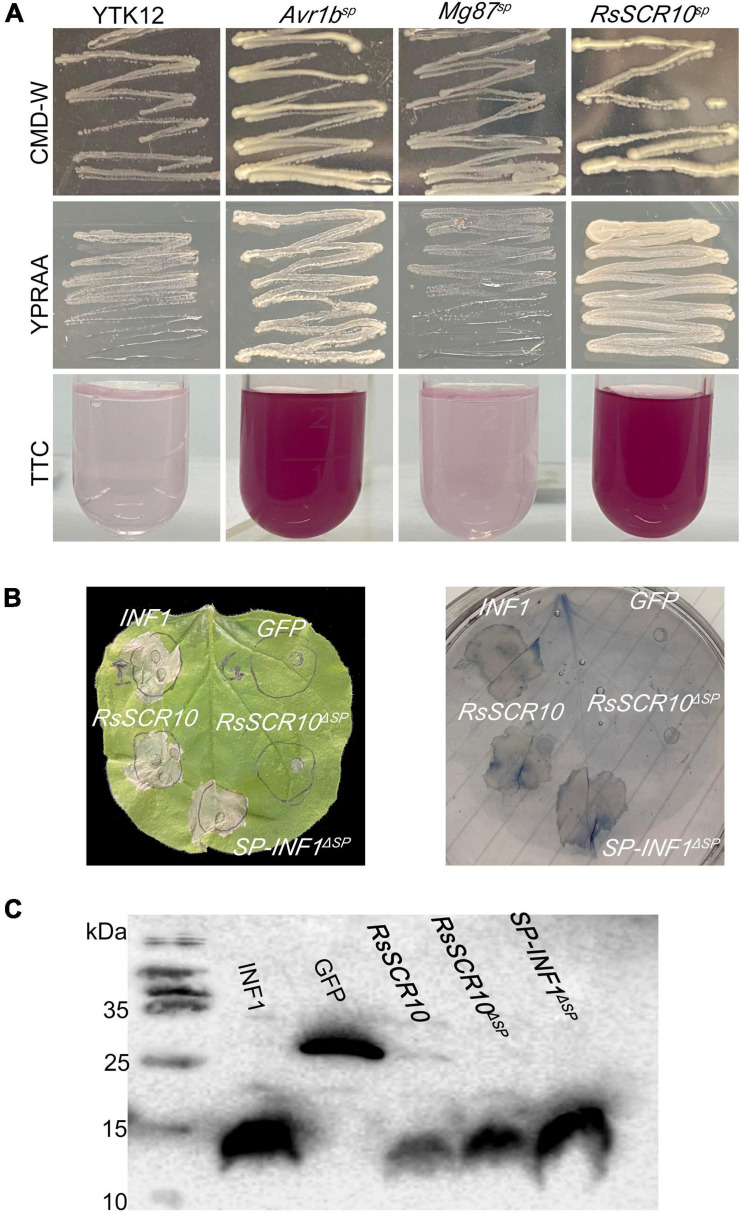
Functional validation of SPs of RsSCR10. **(A)** Yeast invertase secretion assay. YTK12 and Mg87^*sp*^, negative controls; Avr1b^*sp*^, positive control. Color conversion due to the reduction of 2,3,5-triphenyltetrazolium chloride (TTC) to insoluble red-colored 1,3,5-triphenylformazan (TPF) by invertase activity derived from yeast cells was monitored. **(B)** Experiments swapping the RsSCR10 with an SP of INF1. **(C)** Expression of proteins in infiltrated leaves was detected by western blotting.

A probable SP was predicted in the N-terminus of RsSCR10 *via* a search using SignalP 4.1 (Petersen et al., 2011). To validate the function of the SP of RsSCR10, a signal sequence trap system in yeast (Jacobs et al., 1997) was employed. As expected, the fusion protein of invertase with the SP from RsSCR10 was secreted from the transformed yeast YTK12 strain (Figure 2), indicating that RsSCR10 is trafficked *via* the classical secretory pathway. Furthermore, in the agroinfiltration bioassays, there were differences in bioactivity between the full-length RsSCR10 and the partial-length RsSCR10 that lacked the SP. When the SP of elicitin (encoded by the *INF1* gene) was substituted for the SP of RsSCR10, the fusion protein induced cell death in tobacco (Figure 2B). These results indicated that the SP encoded by the gene *RsSCR10* has a secretory function and is necessary for RsSCR10 to induce cell death into tobacco.

### RsSCR10 Triggers Plant Immune Response

**FIGURE 3:**
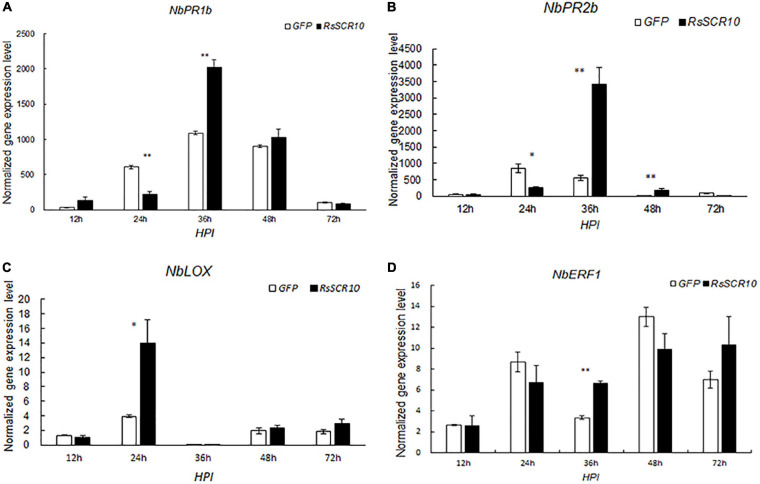
Expression patterns of host defense genes after agroinfiltration of RsSCR10. Transcript levels of the genes **(A)** *NbPR1b*, **(B)** *NbPR2b*, **(C)** *NbLOX*, and **(D)** *NbERF1*, induced by RsSCR10 at different time points. *NbPR1b* and *NbPR2b* are key genes of the salicylic acid pathway, *NbLOX* is a key gene of the jasmonic acid pathway, and *NbERF1* is a key gene of the ethylene pathway. Error bars represent standard errors from three biological replicates. Asterisks indicate statistical significance (Student’s *t*-test) at *5% level and **1% level.

To investigate whether RsSCR10 activates the immune response in *N. benthamiana*, qRT-PCR was used to evaluate the expression of several genes related to activation of the immune response in *N. benthamiana*. Gene markers for host defense signaling, *pathogenesis-related protein 1B* (*PR1b*), *pathogenesis-related protein 2B* (*PR2b*), *ethylene-responsive transcription factor 1* (*ERF1*), and *linoleate 9S-lipoxygenase 5* (*LOX*), which were relevant for salicylic acid, ethylene, and jasmonic acid signaling pathways, respectively (Wang et al., 2000; Lorenzo et al., 2003; Lee et al., 2013), exhibited different expression patterns between agroinfiltrated samples for RsSCR10 and GFP control samples. These differences in gene expression were statistically significant. Specifically, compared with the control samples, *NbPR1b*, *NbPR2b*, and *NbERF1* exhibited higher expression levels at 36 hpi, while *Nb-9-LOX* exhibited higher expression at 24 hpi. These findings support that RsSCR10 has elicitor activity (Figure 3).

### Key Amino Acids of RsSCR10 Induce Cell Death

**FIGURE 4:**
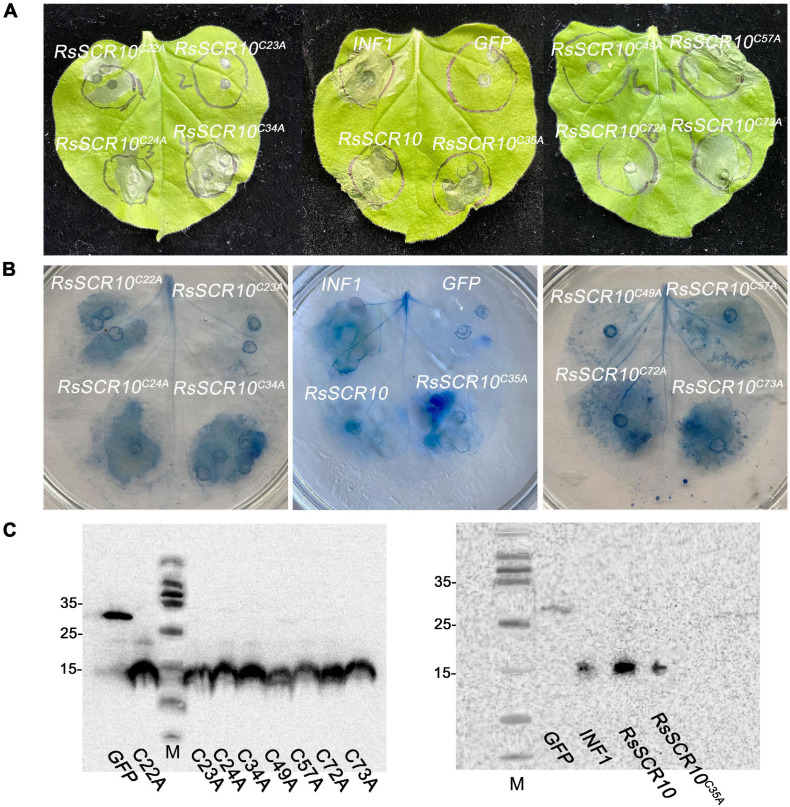
Two cysteine residues of RsSCR10 are required to induce cell death. **(A)** Expression of *RsSCR10* and its mutants constructs in *N. benthamiana* by agroinfiltration. Typical symptoms were photographed at 4 days post-infiltration (dpi). **(B)** Trypan blue staining of *N. benthamiana* leaves. **(C)** Expression of mutant proteins in infiltrated leaves detected by Western blotting. GFP and INF1 were used as negative and positive controls, respectively. Typical symptoms were photographed at 4 dpi. Data are means from three independent experiments.

RsSCR10 encodes an 84-aa protein that contains a predicted N-terminal SP (19 aa) and 10 conserved cysteines (Cys) residues. To identify the key amino acid residues of RsSCR10 that were required for induction of cell death, RsSCR10 sequences alignment in 25 strains of *R. solani* AG1 IA isolated from different areas of China were analyzed (Supplementary Figure 3; Wei et al., 2020). Among the 25 strains, cysteine residues were particularly conserved in RsSCR10 (Supplementary Figure 3). The functionality of Cys residues in RsSCR10 was assessed by mutating Cys sites to Alanine in RsSCR10 using overlap-PCR, and transient expression constructs containing the mutations in RsSCR10 were infiltrated into tobacco leaves. RsSCR10^*C*23*A*^ and RsSCR10^*C*49*A*^ could not induce tobacco cell death (Figure 4A), but cell death was induced in *N. benthamiana* leaves by RsSCR10 containing the other Cys mutations (Figures 4A,B). Western blotting revealed that all mutated proteins of RsSCR10 were expressed in the infiltrated leaves (Figure 4C). These results indicate that mutations of residues 23 and 49 prevented RsSCR10 from triggering cell death in *N. benthamiana* and therefore these sites might be key amino acid residues for the function of RsSCR10.

### RsSCR10 Is Inducible During Infection Onto Host Plants

**FIGURE 5:**
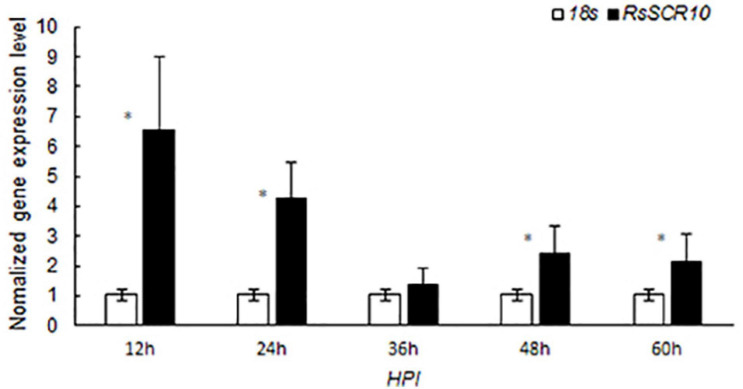
Gene expression patterns of *RsSCR10* after inoculation onto rice variety R600, which is relatively susceptible to rice blast. *R. solani* AG1 IA was used to infect R600, and RsSCR10 expression levels were determined at different time points. Error bars represent standard errors from three biological replicates. *Statistical significance at 5% level (Student’s *t*-test).

Effector genes in filamentous phytopathogens are often transcriptionally induced during infection (Stergiopoulos and De Wit, 2009; Xia et al., 2017). To explore whether RsSCR10 was regulated differently after inoculation onto plant hosts exhibiting variations in sheath blight resistance, gene expression patterns of *RsSCR10* in *R. solani* AG1 IA inoculated on the susceptible rice variety R600, expression of *RsSCR10* was significantly increased at 12, 24, 48, and 60 hpi, relative to the 18S rRNA gene, and the highest expression among the samples analyzed were detected at 12 hpi (at 1% level in Student’s *t*-test) (Figure 5). After inoculating rice leaves with rice sheath blight AG1 IA, a small number of disease spots were found 12 h later. After 24 h, the disease spots increased and gradually expanded into moiré, with gray white spots in the middle. At 60 h, the disease of leaves was serious and becoming yellows. Increased expression of *RsSCR10* at 12 hpi on R600 may be necessary to modulate host immunity. This result shows that RsSCR10 is possibly involved in the preprocess of pathogenesis by *R. solani*.

### RsSCR10 Induction of Cell Death Is Dependent on Hsp90

**FIGURE 6:**
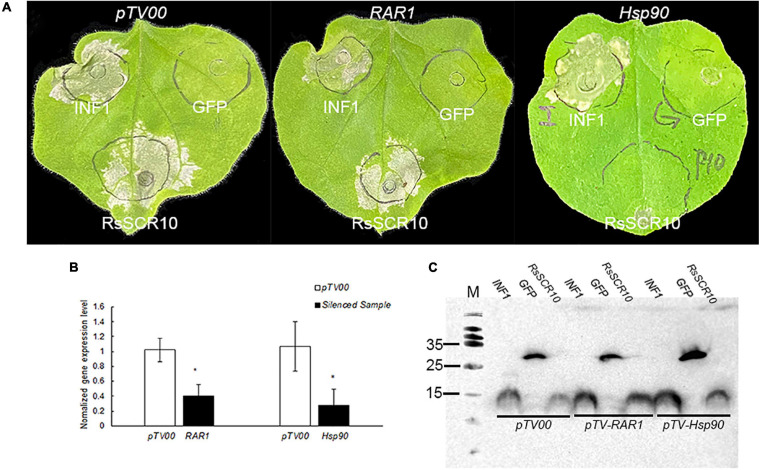
Hsp90 was required for RsSCR10-induced cell death in *N. benthamiana*. **(A)** RsSCR10 was transiently expressed in *N. benthamiana* leaves silenced for pTV00 (control), Hsp90, or RAR1. GFP and INF1 were control proteins. Typical symptoms were photographed 5 days postinfiltration. **(B)** Transcription of genes in silenced *N. benthamiana* measured by qRT-PCR. Error bars represent standard errors from three biological replicates. *Statistical significance at 5% level (Student’s *t*-test). **(C)** Expression of proteins in infiltrated leaves detected by Western blotting. All data are means from three independent experiments.

To explore plant immunological pathways affected by RsSCR10, an independent VIGS assay in tobacco was designed for two genes—*Hsp90*, encoding the molecular chaperone heat shock protein 90 that acts to stabilize R proteins, and *RAR1*, which is required for Mla12 resistance and is involved in PAMP-triggered immunity (PTI), with both genes playing a role in effector-triggered immunity (ETI) (Shirasu, 2003, 2009). Tobacco plants with each gene silenced were agroinfiltrated with RsSCR10 or INF1. RsSCR10 failed to trigger cell death in Hsp90-silenced plants, whereas it induced cell death in RAR1-silenced plants (Figure 6A). Quantitative gene expression analysis confirmed the successful silencing of the two genes (Figure 6B). This was consistent with *RsSCR10* not being a PAMP but being an effector protein. Furthermore, the results suggest that cell death induced by RsSCR10 is dependent on either of the R proteins stabilized by Hsp90 but not by RAR1.

### Translocation Behavior of RsSCR10 in Plant Host Cells

**FIGURE 7:**
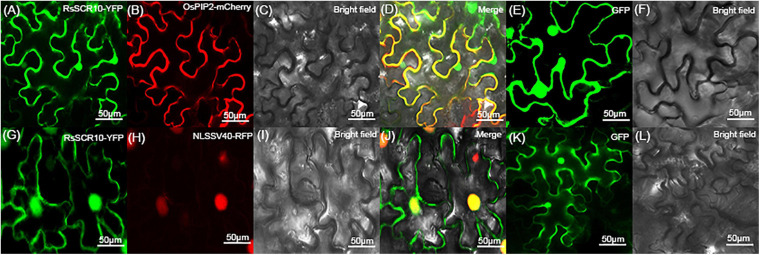
Subcellular localization of RsSCR10 in tobacco epidermal cells. **(A–D)** The fusion protein (RsSCR10-YFP) was coexpressed with the plasma membrane marker (OsPIP2.1-mCherry) (Dangol et al., 2017). **(G–J)** The fusion protein (RsSCR10-YFP) was coexpressed with the nucleus marker (PR82:2 × RFP-NLSSV40) (Huang et al., 2014). **(E,F,K,L)** Subcellular localization of the control (GFP) in tobacco leaf epidermis cells. Images were captured 2–3 days postinfiltration. Bars, 50 μm. All experiments were repeated at least three times.

To determine the subcellular localization of RsSCR10, the protein was explored by transiently expressing 2^×^35S: *RsSCR10*-YFP in tobacco leaf epidermis cells. Transient expression of 2^×^5S: YFP was used as a control. Green fluorescence was observed in multiple subcellular compartments of the infiltrated *N. benthamiana* cells (Figure 7). RsSCR10-YFP was coexpressed with the plasma membrane marker OsPIP2.1-mCherry and the nucleus marker PR82:2 × RFP-NLSSV40, respectively, into tobacco cells. Red fluorescence from OsPIP2.1-mCherry was partially overlapped with green fluorescence, indicating that RsSCR10 is not only localized to the membrane but also in the cytoplasm (Figures 7A–D). Meanwhile, the nucleus marker PR82:2 × RFP-NLSSV40 coexpressed with RsSCR10 further showed that RsSCR10-YFP is present in the nuclei of *N. benthamiana* cells (Figures 7G–J). To investigate whether the same subcellular localization occurred in different plant host cells, transient expression assays were conducted in rice protoplasts using the same YFP fusion constructs. Transient expression of RsSCR10-YFP in rice protoplasts exhibited a similar subcellular localization in the nuclei, cytoplasm, and membrane as well (Supplementary Figure 4).

### RsSCR10 Interacts With Five Rice Proteins

**FIGURE 8:**
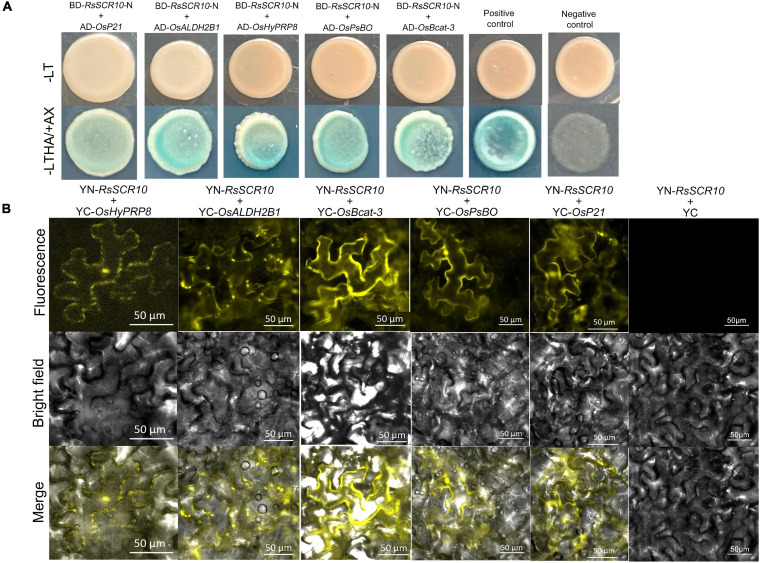
RsSCR10 interacts with five rice proteins. **(A)** Yeast two-hybrid assay showing the interaction of RsSCR10 with five rice proteins. pGBKT7-lam + pGADT7-TB and pGBKT7-53 + pGADT7-T were used as negative and positive controls, respectively. Murine p53 fused to GAL4 DNA BD and the SV40 large T-antigen fused to GAL4 DNA AD were used as the positive control pair. Lamin fused to GAL4 DNA BD and the SV40 large T-antigen fused to GAL4 DNA AD were used as the negative control pair. The N-terminus of RsSCR10 (RsSCR10-N) was used for interaction analysis to exclude the transcriptional activation activity of RsSCR10. **(B)** Bimolecular fluorescence complementation analysis of the interactions between RsSCR10 and five rice proteins. Bars, 50 μm. RsSCR10 fused to the N-terminal fragment of yellow fluorescent protein (YFP) and the C-terminal fragment of YFP were used as the negative control pair (YN-RsSCR10 + YC). All experiments were repeated at least three times.

**FIGURE 9:**
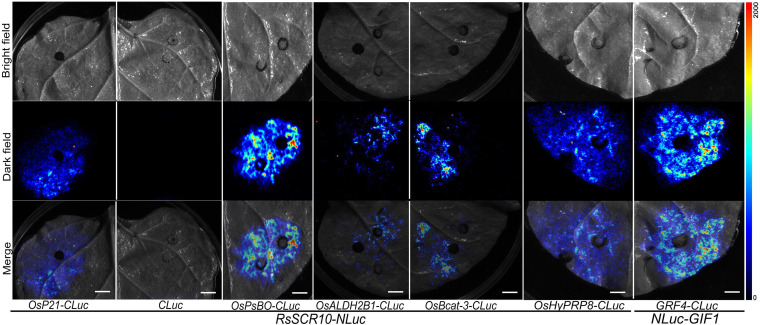
Luciferase complementation assays for RsSCR10 with five rice proteins. The LCA assay in tobacco leaf epidermis cells. NLuc-GIF1 and GRF4-CLuc are positive controls (He et al., 2021). NLuc-RsSCR10 and CLuc are the negative controls. All experiments were repeated at least three times.

Identification of protein cross-interaction between pathogens and plant host organisms could provide a hint of how RsSCR10 modulates host immunity. A yeast two-hybrid system was utilized to screen interacting partners of RsSCR10 from a normalized cDNA library constructed from rice leaf blades at the reproductive stage. Multiple-time screening retained potential positive cDNA clones for 30 proteins. Screening of multiple yeast databases identified 13 positive proteins (positive in at least three repeats) from the 30 genes and the prey and bait plasmids were then co-transformed into the Y2H gold strain. Five positive genes were obtained (Figure 8A), these were type III chlorophyll a/b-binding protein (*OsP21*) [MSU locus ID: LOC_Os07g37550], aldehyde dehydrogenase (*OsALDH2B1*) [MSU locus ID: LOC_Os06g15990], photosystem II subunit PsbO (*OsPsbO*) [MSU locus ID: LOC_Os01g31690], hybrid proline- or glycine-rich protein 8 (*OsHyPRP8*) ([MSU locus ID: LOC_ Os04g46830], and similar to branched-chain-amino-acid aminotransferase 3 (*OsBcat-3*) [MSU locus ID: LOC_Os03g12890]. Bimolecular fluorescence complementation (BiFC) assays in tobacco epidermal cells and luciferase complementation assays (LCAs) in tobacco leaves confirmed the cross-interactions of the above five proteins with RsSCR10 (Figures 8B, 9). These results proved that these five proteins are probable interacting partners of RsSCR10 in host plant cells. Moreover, the results indicate that the RsSCR10 is instrumental in the process of host infection and may interfere with the growth and development of the host, which is conducive to the invasion of pathogenic fungi.

## Discussion

Critical molecular functions of SCR proteins in pathogenesis have recently been emphasized in various phytopathogens (Chen et al., 2014, 2016; Nguyen et al., 2018). In the present study, an SCR-secreted protein of sheath blight phytopathogen *R. solani* was observed to display elicitor activity to tobacco and *Arabidopsis* plants. The ability of RsSCR10 to activate the *N. benthamiana* immune response was demonstrated by confirming the expression of PR genes, the activation of H_2_O_2_, and callose deposition. Thus, RsSCR10 activated the *N. benthamiana* immune system. Cys residues in SCR proteins might offer disulfide bonds to protect against biochemical degradation in the host apoplast (Stergiopoulos and De Wit, 2009). As the results show that mutations at two cysteine sites of the RsSCR10 caused RsSCR10 to lose its function of induced cell death. Mutations of these two amino acid sites (residues 23 and 49) may therefore destroy the stable structure of the RsSCR10 protein, thus the function is lost. The small cysteine-rich protein RsSCR10 carries a predicted N-terminal SP for secretion. The prediction was supported by the yeast secretion assay, in which the putative SP of RsSCR10 was functional to guide the secretion of the truncated invertase (Figure 2). In addition, RsSCR10-triggered cell death in *N. benthamiana* was dependent on the SP of RsSCR10 (Figure 2B). The effector RsIA_NP8 in *R. solani* AG1 IA also requires its predicted SP to induce cell death (Wei et al., 2020).

The effector protein is secreted by the pathogen to the cell interaction interface when it infects the plant. After breaking through the plant epidermis, they release two types of SCR effector proteins to perform functions in the ectoplasmic space of the host cell or the cell (Dodds and Rathjen, 2010). It is well known that the intracellular effectors of plant filamentous pathogens are presumably secreted into extracellular spaces under the guide of N-terminal SPs before being transited into the host cells (Dou and Zhou, 2012). The cytoplasmic effectors in *M. oryzae*, such as AvrPiz-t and PWL2, are accumulated in the biotrophic interfacial complex (BIC) during infection and are then translocated into plant cells (Khang et al., 2010; Park et al., 2012). The cytoplasmic effectors SCRE2 in *Ustilaginoidea virens* guided by its SP into the plant cell to perform its function as well (Fang et al., 2019). Confocal microscopy showed that RsSCR10-YFP was localized intracellularly when RsSCR10-YFP was transiently expressed in rice protoplasts and *N. benthamiana* leaves (Figure 7 and Supplementary Figure 4). Hence, the SP of RsSCR10 might have the function of transferring RsSCR10 from the apoplast to intracellular. However, the precise localization of RsSCR10 needs further elucidation, such as the subsequent plasmolysis experiment that can be used to detect the presence of RsSCR10 fluorescent protein in the apoplast space of plant cells to further determine the influence of SP on the localization of RsSCR10.

In the VIGS assays, cell death by RsSCR10 was dependent on Hsp90 (Figure 6), a host protein required for R protein-mediated inflammasome activation. However, the function of Hsp90 is not limited to R protein family members (Ye and Ting, 2008). Shirasu (2009) reported that SGT1 was a co-chaperone of Hsp90, and the two molecules form a chaperone complex. Based on this evidence, the Hsp90 and SGT1 could act to stabilize a protein in the cell death signaling pathway. It is not known whether Hsp90 and SGT1 stabilize a receptor for RsSCR10 or not, but it remains a possibility.

Necrotrophic phytopathogens attempt to neutralize host plant immune reactions by releasing molecular proteins including effector proteins (Weiberg et al., 2013; Zhu et al., 2013; Takahara et al., 2016). Furthermore, attacked plant cells express inductive immunity to defend against such phytopathogens. Recent reports have highlighted the critical roles of chloroplasts in plant immunity (Sano et al., 2014; Jarvi et al., 2016; Serrano et al., 2016), and this may indicate that the production of defense-related signaling molecules affects plant immunity. Plant photosynthesis is a core fundamental physiology that drives not only plant vegetative growth and reproduction but also protection (Karpinski et al., 2013; Zhou et al., 2015). RsSCR10 interacted with five rice proteins, and the protein products encoded by those genes were highly expressed in rice leaves and roots. The chloroplast is the location of photosynthesis in higher plants, and this organelle plays a vital role in plant growth and development. When a plant is infected with a virus, the virus will use the chloroplast for replication and propagation; destroy structural elements related to the chloroplast; affect the photosystem, electron transport chain, and other processes; and ultimately affect the normal growth of the plant (Zhao et al., 2016). The protein interaction verification experiments in the current study proved a potential interaction between RsSCR10 and several photosynthetic proteins such as type III chlorophyll a/b-binding protein and OsPsbO, implying veiled protein cross-interactions with biochemical selectivity in chloroplasts. Chloroplasts have a central role in plant immunity by hosting the biosynthesis of several key defense-related molecules, including hormones and secondary messengers such as calcium and reactive oxygen species (ROS) (Serrano et al., 2016; Ghosh et al., 2017). However, based on the current experimental results, further experiments are needed to verify the interaction relationship between RsSCR10 and 5 rice proteins, such as *in vivo* interaction verification experiment, co-immunoprecipitation (CoIP), or *in vitro* GST pull-down experiment to verify the interaction.

Induction of cell death in tobacco leaves by RsSCR10 might be one aspect of the pathogenic strategy of *R. solani*. Results from the current study acknowledged that the elicitor activity of RsSCR10 was sufficient in the bioassay. However, the exact role RsSCR10 plays in necrosis has yet to be elucidated. Furthermore, many genes encoding SCR proteins were predicted in phytopathogenic genomes (Saunders et al., 2012; Zheng et al., 2013), and these warrant exploration in the future. Overall, the detailed molecular mechanisms of how RsSCR10 participates in rice–*R. solani* interactions require further investigation.

## Data Availability Statement

The original contributions presented in the study are included in the article/Supplementary Material, further inquiries can be directed to the corresponding author/s.

## Author Contributions

XN and GY designed this study. XN wrote the manuscript. XN, GY, and HL conducted the experiments. XN and YL assisted the experimental analyses. YL gave the common vector sequence and constructed part of the VIGS vector. AZ and PL organized this study. All authors reviewed the manuscript.

## Conflict of Interest

The authors declare that the research was conducted in the absence of any commercial or financial relationships that could be construed as a potential conflict of interest.

## Publisher’s Note

All claims expressed in this article are solely those of the authors and do not necessarily represent those of their affiliated organizations, or those of the publisher, the editors and the reviewers. Any product that may be evaluated in this article, or claim that may be made by its manufacturer, is not guaranteed or endorsed by the publisher.

## Footnotes

1
http://www.R-project.org/

## Supplementary Material

Supplementary Figure 1 RsSCR10 triggers plant immunity responses in *N. benthamiana*. **(A)** Cell death in *N. benthamiana* leaves indicated by trypan blue staining. GFP and Bax were used as negative and positive controls, respectively. Typical symptoms were photographed at 4 days postinfiltration. **(B)** Accumulation of ROS and deposition of callose in *N. benthamiana*. For observation of callose, bars = 100 μm. Experiments were replicated at least three times.

Supplementary Figure 2 RsSCR10 induced upregulated expression of genes related to H_2_O_2_ synthesis in *N. benthamiana*. Asterisks indicate statistical significance (Student’s *t*-test) at *5% level and **1% level. Error bars represent standard errors from three biological replicates.

Supplementary Figure 3 Sequence alignment of RsSCR10 in 25 strains of *R. solani* AG1 IA.

Supplementary Figure 4 RsSCR10 localization analysis in rice protoplasts. **(A–D)** The fusion protein (RsSCR10-YFP) was coexpressed with the plasma membrane marker (OsPIP2.1-mCherry) (Dangol et al., 2017). **(G–J)** The fusion protein (RsSCR10-YFP) was coexpressed with the nucleus marker (PR82:2 × RFP-NLSSV40) (Huang et al., 2014). **(E,F,K,L)** Subcellular localization of the control (GFP) in rice protoplasts. Fluorescence was observed after incubation for 3–16 h. Bars, 50 μm. All experiments were repeated at least three times.

Supplementary Table 1 Primer sequences used in this study.
