# Supplementary figures and images for: A Novel, Small Cysteine-Rich Effector, RsSCR10 in Rhizoctonia solani Is Sufficient to Trigger Plant Cell Death
Source: Front Microbiol. 2021 Aug 23;12:684923. doi: 10.3389/fmicb.2021.684923 (PMC8421026; doi:10.3389/fmicb.2021.684923)

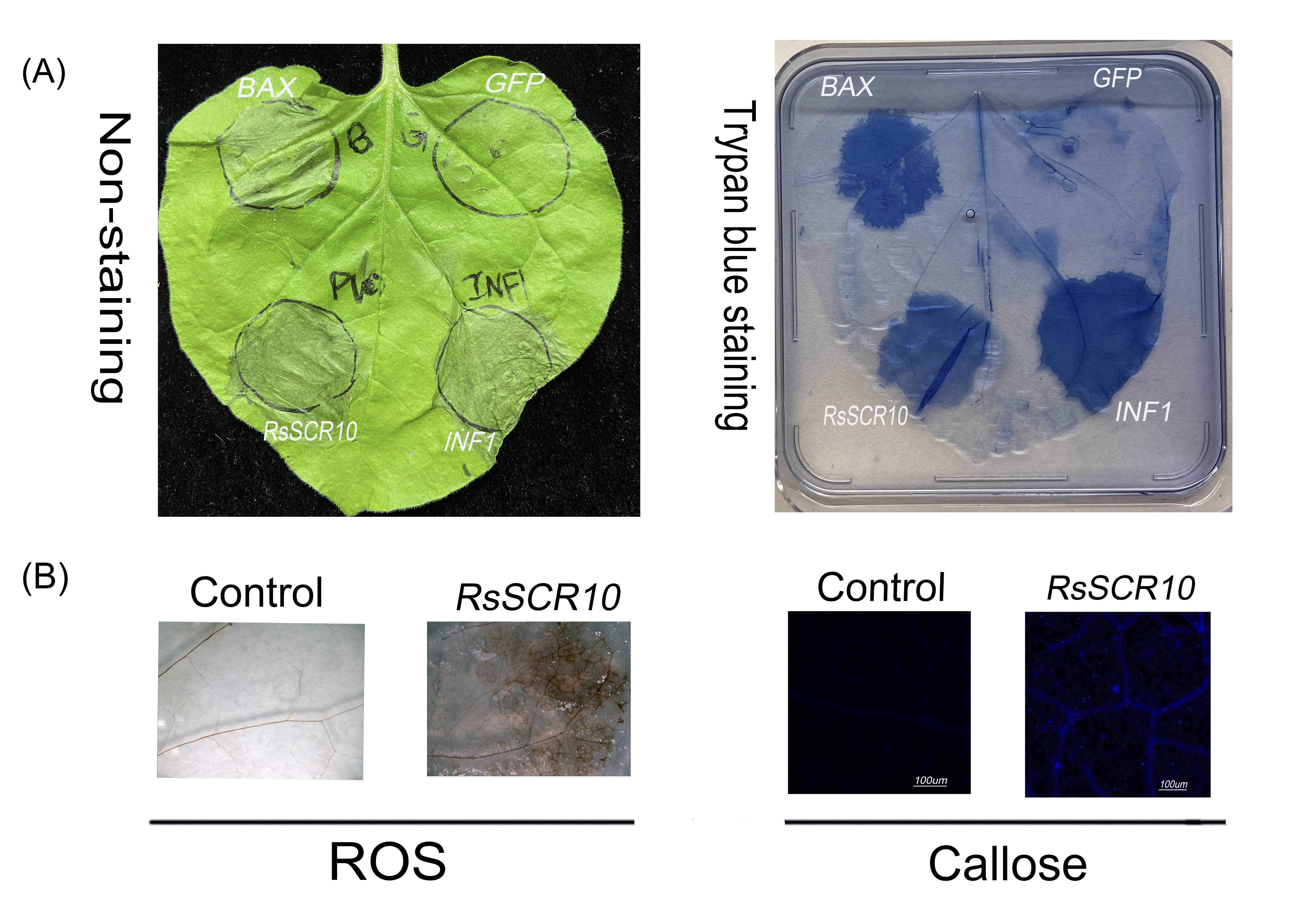

Supplement: Supplementary Figure 1 — RsSCR10 triggers plant immunity responses in N. benthamiana. (A) Cell death in N. benthamiana leaves indicated by trypan blue staining. GFP and Bax were used as negative and positive controls, respectively. Typical symptoms were photographed at 4 days postinfiltration. (B) Accumulation of ROS and deposition of callose in N. benthamiana. For observation of callose, bars = 100 μm. Experiments were replicated at least three times. [file Image_1.JPEG]

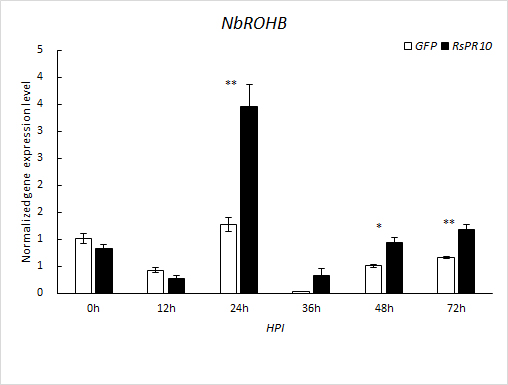

Supplement: Supplementary Figure 2 — RsSCR10 induced upregulated expression of genes related to H2O2 synthesis in N. benthamiana. Asterisks indicate statistical significance (Student’s t-test) at *5% level and **1% level. Error bars represent standard errors from three biological replicates. [file Image_2.JPEG]

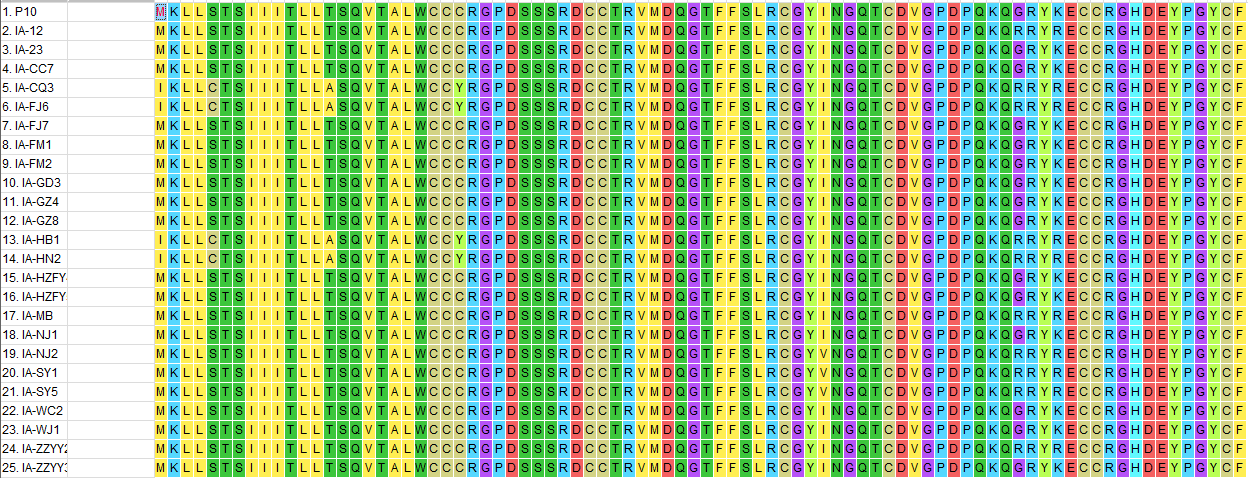

Supplement: Supplementary Figure 3 — Sequence alignment of RsSCR10 in 25 strains of R. solani AG1 IA. [file Image_3.PNG]

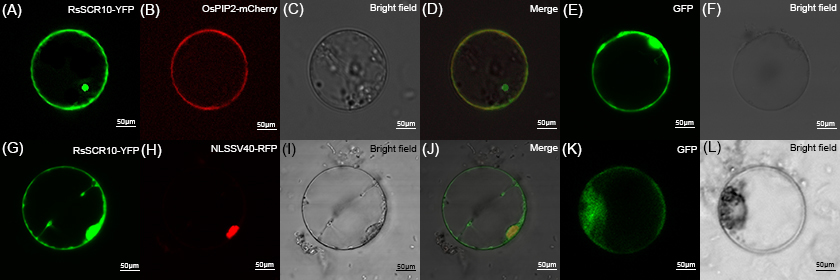

Supplement: Supplementary Figure 4 — RsSCR10 localization analysis in rice protoplasts. (A–D) The fusion protein (RsSCR10-YFP) was coexpressed with the plasma membrane marker (OsPIP2.1-mCherry) (Dangol et al., 2017). (G–J) The fusion protein (RsSCR10-YFP) was coexpressed with the nucleus marker (PR82:2 × RFP-NLSSV40) (Huang et al., 2014). (E,F,K,L) Subcellular localization of the control (GFP) in rice protoplasts. Fluorescence was observed after incubation for 3–16 h. Bars, 50 μm. All experiments were repeated at least three times. [file Image_4.JPEG]
